# KDM6A addiction of cervical carcinoma cell lines is triggered by E7 and mediated by p21^CIP1^ suppression of replication stress

**DOI:** 10.1371/journal.ppat.1006661

**Published:** 2017-10-02

**Authors:** David R. Soto, Christopher Barton, Karl Munger, Margaret E. McLaughlin-Drubin

**Affiliations:** 1 Infectious Diseases Division, Department of Medicine, The Channing Laboratory, Harvard Medical School, Boston, Massachusetts, United States of America; 2 Department of Developmental, Molecular, and Chemical Biology, Tufts University School of Medicine, Boston, Massachusetts, United States of America; University of Wisconsin-Madison, UNITED STATES

## Abstract

Expression of E7 proteins encoded by carcinogenic, high-risk human papillomaviruses (HPVs) triggers increased expression of the histone H3 lysine 27 demethylase KDM6A. KDM6A expression is necessary for survival of high-risk HPV E7 expressing cells, including several cervical cancer lines. Here we show that increased KDM6A in response to high-risk HPV E7 expression causes epigenetic de-repression of the cell cycle and DNA replication inhibitor p21^CIP1^, and p21^CIP1^ expression is necessary for survival of high-risk HPV E7 expressing cells. The requirement for KDM6A and p21^CIP1^ expression for survival of high-risk HPV E7 expressing cells is based on p21^CIP1^’s ability to inhibit DNA replication through PCNA binding. We show that ectopic expression of cellular replication factors can rescue the loss of cell viability in response to p21^CIP1^ and KDM6A depletion. Moreover, we discovered that nucleoside supplementation will override the loss of cell viability in response to p21^CIP1^ depletion, suggesting that p21^CIP1^ depletion causes lethal replication stress. This model is further supported by increased double strand DNA breaks upon KDM6A or p21^CIP1^ depletion and DNA combing experiments that show aberrant re-replication upon KDM6A or p21^CIP1^ depletion in high-risk HPV E7 expressing cells. Therefore, KDM6A and p21^CIP1^ expression are essential to curb E7 induced replication stress to levels that do not markedly interfere with cell viability.

## Author summary

High-risk human papillomaviruses (HPVs) are associated with approximately five percent of all human cancers, including virtually all cervical cancers as well as a large percentage of anal, vaginal, vulvar, penile, and oropharyngeal cancers. The HPV E6 and E7 proteins are the major oncogenic drivers in these tumors, and persistent expression of E6 and E7 is required for the maintenance of the transformed state. While E6 and E7 lack intrinsic enzymatic activities, and thus are difficult to directly target therapeutically, they biochemically interact with, functionally modify, or alter expression of key host cellular signaling proteins. HPV16 E7 triggers increased expression of the KDM6A histone demethylase, and KDM6A expression becomes necessary for the survival of HPV16 E7 expressing cells. Here we show that the requirement for persistent KDM6A expression is mediated by the cell cycle and DNA replication inhibitor p21^CIP1^ in that p21^CIP1^ expression is necessary for survival of E7 expressing cells. Remarkably, this is based on the ability of p21^CIP1^ to inhibit cellular DNA replication by binding PCNA. Our results suggest that increased KDM6A and p21^CIP1^ expression serves to curb HPV16 E7-induced replication stress to levels that are conducive to DNA replication but do not cause death of HPV infected cells.

## Introduction

Human papillomaviruses (HPVs) are a group of small, double-stranded DNA viruses that infect the squamous epithelium. The more than 200 HPV types described to date can be divided into mucosal and cutaneous types based on their tissue tropism. The mucosal HPVs can be clinically designated “low-risk” or “high-risk” based on their propensity to cause lesions that can undergo malignant progression. High-risk HPV infections account for approximately 5% of all human cancers, most notably cervical carcinomas, the third most common cancer in women worldwide [1, 2]. Other anogenital tract cancers, including anal, vulvar, vaginal, and penile cancers, as well as oropharyngeal cancers, are also frequently associated with high-risk HPV infections [3, 4]. The currently available prophylactic vaccines have no therapeutic efficacy. In addition, HPV-associated cervical cancers arise years to decades after the initial infection and vaccination rates remain low in many countries; as such, it will be decades before the current vaccination efforts will have a measurable impact on the incidence of HPV-associated tumors [5].

The E6 and E7 proteins are the major drivers of HPV-associated cancers, and persistent E6 and E7 expression is necessary for the survival of these tumors. E6 and E7 encode small non-enzymatic proteins that drive cancer formation by functionally re-programming cellular signal transduction pathways. The best known cellular targets of high-risk mucosal HPV E6 and E7 proteins are the p53 and retinoblastoma (pRB) tumor suppressors, respectively. Notably, these tumor suppressor pathways are also rendered dysfunctional by mutation in almost all human solid tumors [6, 7]. Amongst the additional cellular targets of the HPV E6 and E7 oncoproteins that have been identified are enzymes that modulate histone modifications [8–17]. Dynamic post-translational modifications of histone tails impact both the physical state and the transcriptional competence of chromatin and play a critical role in the regulation of a variety of cellular processes such as stem cell maintenance, cell fate determination and maintenance, cell cycle control, and epigenetic heritability of transcriptional programs [reviewed in 18, 19]. We previously reported that the repressive trimethylation of lysine 27 on histone H3 (H3K27me3), which is critical for epigenetic silencing mediated by polycomb group (PcG) proteins [20, 21] is dramatically reduced in HPV16 E7-expressing primary human keratinocytes and in HPV16-positive cervical lesions and cancers [15, 17]. The H3K27me3 mark is written by the histone lysine methyltransferase KMT6 (EZH2) subunit of polycomb repressive complex 2 (reviewed in [22]) and erased by the histone lysine demethylases KDM6A (UTX) and KDM6B (JMJD3) [23–27], which are expressed at higher levels in these cells [15, 17].

Although KDM6A and KDM6B appear identical with regards to catalytic activities and histone substrate specificities, KDM6A and KDM6B have non-overlapping and non-redundant biological activities. KDM6B may have both tumor suppressive and oncogenic activities in different cancer types. The *KDM6B* gene is located at *5q31*, an area that is frequently lost in various malignancies, including myeloid leukemias. However, KDM6B is expressed at high levels in prostate cancer, and its expression is further increased in metastatic prostate cancer [28]. Similarly, KDM6A appears to also have both tumor suppressive and oncogenic activities. Inactivating somatic *KDM6A* mutations have been detected in multiple cancers, including medulloblastoma, multiple myeloma, esophageal carcinomas, renal cell carcinoma, bladder cancer, and prostate tumors [29–31]. In contrast, KDM6A is rarely mutated in breast tumors, and activates oncogenic gene expression programs that control proliferation and invasion [32, 33]. In a study of over 800 cervical and head and neck tumors from The Cancer Genome Atlas (TCGA), HPV-positive tumors were found to express higher levels of KDM6A [34]. Moreover, KDM6B, but not KDM6A, regulates RAS/RAF-mediated oncogene-induced senescence (OIS), one of several cell-intrinsic tumor-suppressor responses that function to eliminate aberrantly proliferating, potentially premalignant cells [35, 36]. OIS is a major barrier to malignant progression, and additional genetic or epigenetic alterations are needed for progression to invasive cancer [37].

HPV16 E7 expression causes increased expression of both KDM6A and KDM6B, and HPV16 E7 expressing cells are dependent on KDM6A and KDM6B expression for cell survival [14, 15]. Our previous studies revealed that the p16^INK4A^ tumor suppressor is a critical downstream transcriptional target of KDM6B, and that p16^INK4A^ expression is necessary for viability of high-risk HPV expressing cells [14].

Here we report that KDM6A expression is stimulated by high-risk, but not low-risk, HPV E7 proteins and that KDM6A expression is necessary for viability of high-risk, but not low-risk, HPV expressing or normal cells. We show that KDM6A controls expression of the cell cycle and replication inhibitor, p21^CIP1^, and that KDM6A mediated induction of p21^CIP1^ expression is necessary for viability of high-risk HPV E7 expressing cells. We find that the ability of p21^CIP1^ to bind PCNA and inhibit DNA replication, rather than CDK2 binding and inhibition, is critical. Overall, our results suggest a model whereby p21^CIP1^ expression is necessary for the viability of HPV16 E7 expressing cells by dampening E7-induced replication stress.

## Results

### High-risk, but not low-risk, HPV E7 trigger increased KDM6A expression

**Fig 1:**
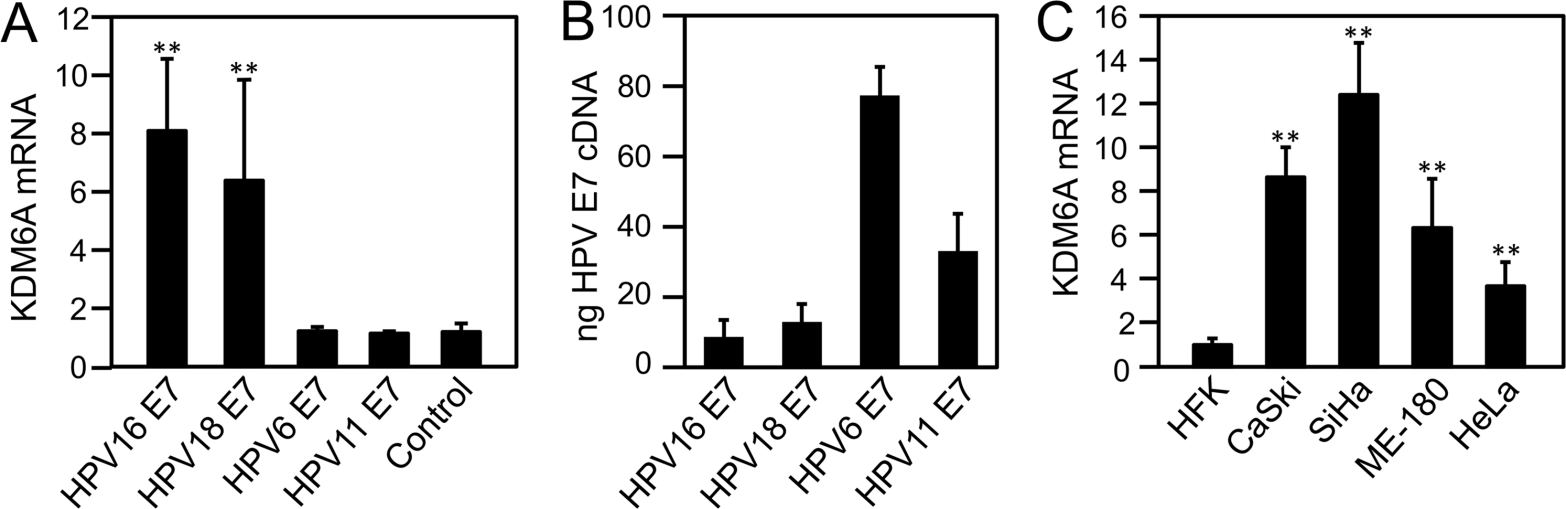
High-risk, but not low-risk HPV E7 proteins induce KDM6A expression. *(A)* Quantitative real-time RT-PCR analysis of KDM6A mRNA expression in HFK and HFK/E7 cells. *(B)* Absolute quantification of HPV E7 mRNA expression in HFK and HFK/E7 cells. *(C)* Quantitative real-time RT-PCR analysis of KDM6A mRNA expression in HFK cells, the HPV16 positive CaSki and SiHa cervical carcinoma cell lines, the HPV39 positive cervical cancer cell line Me-180, and the HPV18 positive cervical cancer cell line HeLa. The bar graphs show averages and SDs from three independent experiments, each performed in triplicate. Increases in HPV16 and 18 E7 expressing cells and the HPV positive CaSki, SiHa, Me-180, and HeLa cells are statistically significant (**), with *P* values < 0.01.

It has been observed that KDM6A expression is increased in HPV16 E7 expressing cells [15, 17]. To determine whether the ability to increase KDM6A expression was shared with other high-risk or low-risk HPV E7 proteins, we compared KDM6A levels in high-risk HPV16 E7 and HPV18 E7 expressing HFKs to HFKs expressing low-risk HPV6 or HPV11 E7. HPV18 E7 expressing HFKs expressed KDM6A at similar levels as HPV16 E7 expressing cells, whereas low-risk HPV6 and HPV11 E7 expressing cells expressed KDM6A at levels similar to control vector transduced cells (Fig 1A). The mRNA levels of the low-risk HPV E7s were higher than high-risk HPV E7s (Fig 1B), and hence the ability of HPV E7 proteins is not related to expression levels but is a unique activity of the high-risk HPV E7 proteins. We next assessed KDM6A levels in several cervical cancer cell lines. KDM6A levels were higher than in primary human foreskin keratinocytes in the HPV16-positive SiHa and CaSki, the HPV18-positive HeLa, and the HPV39-positive Me-180 cervical cancer cell lines (Fig 1C). Thus, KDM6A levels are generally higher in cervical cancer cells and cells expressing high-risk HPV E7 than in normal epithelial cells or cells expressing low-risk HPV E7 proteins.

### KDM6A expression is required for the viability of cervical cancer cells

**Fig 2:**
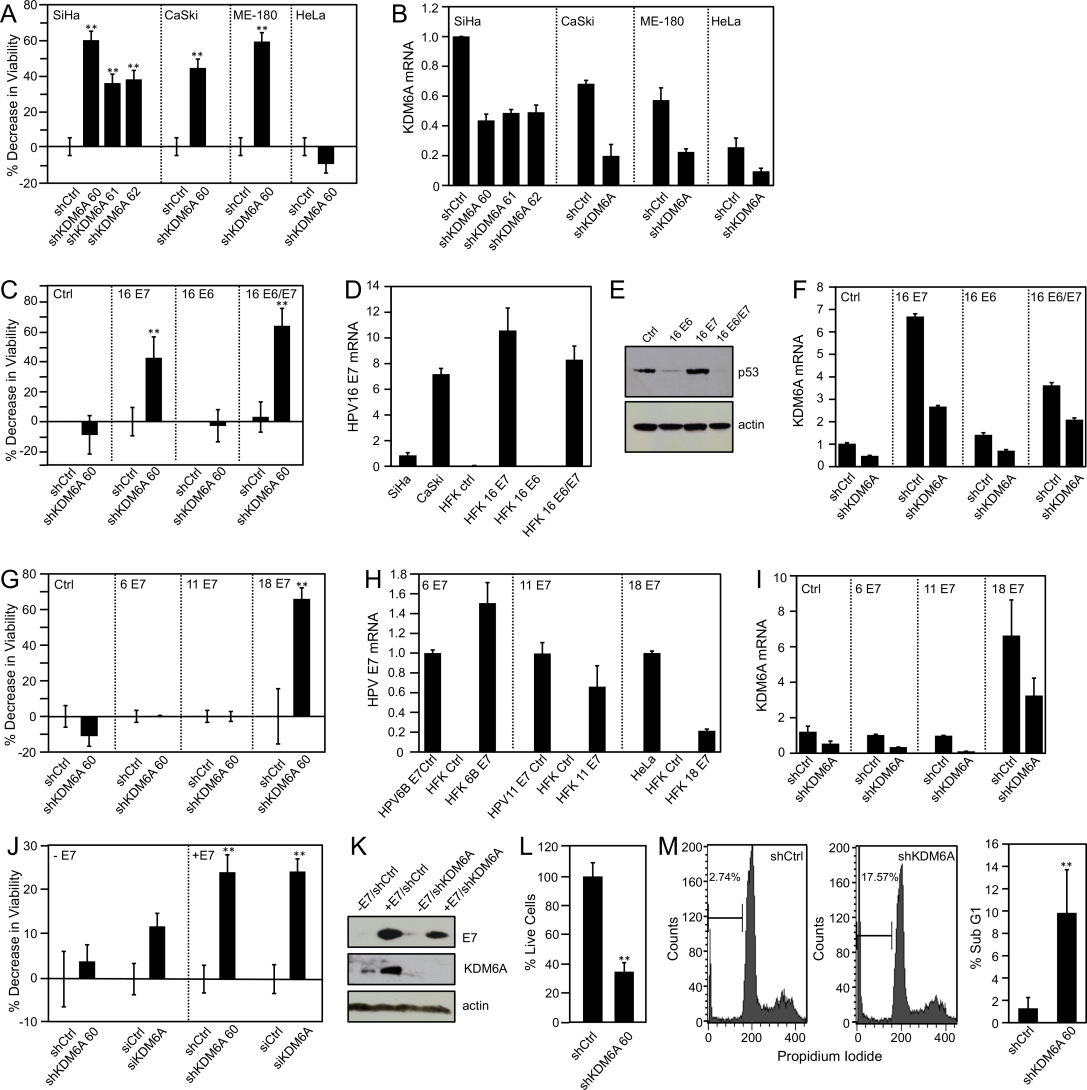
KDM6A addiction is caused by the HPV E7 protein. *(A*,*B)* KDM6A was depleted in the HPV16 positive SiHa and CaSki cervical carcinoma cell lines, the HPV39 positive cervical cancer cell line Me-180, and the HPV18 positive cervical cancer cell line HeLa; *(A)* Cell viability was measured by reduction of resazurin. Three independent KDM6A shRNA constructs (60, 61, and 62) were used in the initial experiments in SiHa cells; *(B)* KDM6A depletion was verified by quantitative real-time RT-PCR; *(C-F)*. KDM6A was depleted in HFKs expressing control vector, HPV16 E7, E6, or E6 and E7; *(C)* Cell viability was measured by reduction of resazurin; *(D)* HPV E7 expression was verified by quantitative real-time RT-PCR and compared to levels found in SiHa and CaSki cervical carcinoma cells; *(E)* Due to the absence of appropriate antibodies, HPV16 E6 expression was determined by assessing p53 levels, which are decreased in HPV16 E6-expressing cells because of E6-mediated proteasomal degradation [66], by Western blot analysis of p53. Lysates were separated by SDS/PAGE, transferred, and probed for p53. An actin blot is included as a loading control; *(F)* KDM6A depletion was verified by quantitative real-time RT-PCR; *(G-I)* KDM6A was depleted in HFKs expressing HPV6, HPV11, or HPV18 E7; *(G)* Cell viability was measured by reduction of resazurin; *(H)* HPV6 E7, HPV11 E7, and HPV18 E7 mRNA levels were determined by qRT-PCR; *(I)* KDM6A depletion was verified by quantitative real-time RT-PCR; *(J*,*K)* KDM6A was depleted in U2OS-tet on cells with doxycycline-inducible expression of HPV16 E7 using a KDM6A shRNA construct or KDMA6A-specific siRNA duplexes. *(J)*. Cell viability was measured by reduction of resazurin; *(K)* Western blot analysis of HPV16 E7 and KDM6A. Lysates were separated by SDS/PAGE, transferred, and probed for HPV16 E7 and KDM6A. An actin blot is included as a loading control; *(L)* KDM6A was depleted in the HPV16 positive SiHa cervical carcinoma cell line. After 10 days of puromycin selection, live cells were stained with sulforhodamine B; and *(M)* Cell cycle profile determined by fluorescence-activated sorting from a representative experiment and percentage of sub-G1 phase from three independent experiments. Averages and SDs for three independent experiments are shown. Statistically significant changes are indicated, **P < 0.01.

We have previously reported that depletion of KDM6A in the HPV16-positive cervical cancer line CaSki markedly inhibited cell viability [15]. To determine whether other cervical carcinoma cells showed a similar phenotype, we depleted KDM6A in the HPV16 positive cervical cancer lines SiHa and CaSki, the HPV39 positive cervical cancer cell line Me-180, and the HPV18 positive cervical cancer cell line HeLa by transfection of a KDM6A-specific shRNA. Three different KDM6A shRNA expression vectors were used initially in SiHa cells (Fig 2A), and shRNA 60 was chosen for the future experiments. KDM6A depletion was verified by qRT-PCR (Fig 2B), and cell viability was assayed three days post-transfection. Consistent with our previously published results [15], KDM6A depletion significantly decreases viability of SiHa cells, ranging from 36%; *P* = 0.0009 to 60%; *P* < 0.0001. Similarly, KDM6A depletion in CaSki and Me-180 cells also caused significant 44% (*P* = 0.0071) and 59% (*P* < 0.0001) decreases in cell viability, respectively. In contrast, KDM6A depletion in HeLa cells caused a minor but non-significant 10% (*P* = 0.1297) increase in cell viability, much like what we previously observed upon KDM6B depletion in this cell line [14]. (Fig 2A). These results indicate that KDM6A expression is necessary for viability of some cervical carcinoma lines. The critical dependence of the viability of cancer cells on specific signaling pathways is generally referred to as “oncogene addiction” or “pathway addiction” [38]. By this definition, cervical carcinoma cells are addicted to KDM6A expression.

### HPV16 E7 expression is sufficient to generate KDM6A addiction

The cervical carcinoma lines tested above contain integrated HPV genomes and consistently express the E6 and E7 oncoproteins. Given that HPV16 E7 causes increased KDM6A expression, we next determined whether HPV E7 oncoprotein expression was sufficient to generate KDM6A addiction. To test this hypothesis, we engineered donor- and passage-matched primary human foreskin keratinocyte (HFK) populations with ectopic expression of high-risk HPV16 E6 and/or E7 or HPV18 E7 and low-risk HPV6 or HPV11 E7 using retroviral vectors and verified expression by qRT-PCR and Western blot (Fig 2D and 2E). KDM6A was depleted by infection with a lentiviral shRNA expression vector, depletion was verified by qRT-PCR (Fig 2F), and cell viability was assessed. We observed a significant 43% (*P* = 0.0052) decrease in cell viability in HPV16 E7-expressing HFKs. Similarly, cell viability was also significantly decreased by 64% (*P* <0.0001) in cells that, like cervical carcinoma cells, co-express HPV16 E6 and E7. In contrast, HPV16 E6-expressing HFKs and control-vector–infected HFK populations were not significantly affected (P = 0.6767 and *P* = 0.1824, respectively) by KDM6A depletion (Fig 2C). KDM6A depletion in HPV18 E7 expressing HFKs caused a significant 65% (*P* = 0.0002) decrease in cell viability, while HPV6 and HPV11 E7 expressing HFKs were not significantly inhibited *(P* = 0.9206 and 0.9863, respectively) (Fig 2G, 2H and 2I). In addition to cell viability assays, we also determined cell numbers by Sulforhodamine B (SRB) assays following infection of SiHa cervical carcinoma cells with the recombinant KDM6A shRNA 60 expressing lentivirus at ten days after puromycin selection (Fig 2L). These experiments revealed that cell numbers were significantly decreased by 65.4% (*P* <0.0001). Moreover, FACS analysis of SiHa cervical cancer cells showed a significant 17.6% (*P* = 0.0003) increase in cells with a sub G0/G1 DNA content, supporting the notion that KDM6A depletion in SiHa cells causes apoptotic cell death (Fig 2M). In summary, these results show that high-risk HPV E7 expression is sufficient to cause KDM6A addiction. In contrast, low-risk HPV E7 expression, which does not trigger KDM6A expression, does not generate KDM6A addiction. Since E6 does not markedly modulate sensitivity to KDM6A depletion, and given that E6 and E7 are the only HPV proteins consistently expressed in cervical carcinoma lines, these results show that KDM6A addiction of cervical carcinoma cells arises as a consequence of E7 expression.

### KDM6A addiction arises as an immediate consequence of HPV16 E7 expression

To determine whether KDM6A addiction is generated as an immediate consequence of HPV16 E7 expression or whether it is acquired after long-term E7 expression, we performed KDM6A depletion experiments in U2OS osteosarcoma cells with doxycycline-inducible HPV16 E7 expression. We showed previously that these cells express HPV16 E7 and KDM6A with a concomitant decrease of the H3K27me3 mark within 48 to 72 hours of doxycycline treatment. This is reversed when doxycycline is removed [15]. Depletion of KDM6A with either shRNA or siRNA duplexes did not significantly inhibit the viability of these cells before HPV16 E7 induction (*P* = 0.1455 and 0.053, respectively). In contrast, KDM6A depletion with shRNA or siRNA duplexes caused a significant 24% (*P* = 0.0043 and 0.0003, respectively) decrease in viability after HPV16 E7 expression was induced by 72 h of doxycycline treatment (Fig 2J). Depletion of KDM6A was verified by immunoblot (Fig 2K). This result shows that KDM6A addiction arises as a direct and immediate consequence of HPV16 E7 expression. This finding is particularly remarkable because, unlike HPV-expressing cervical cancer cells, HPV E7 expression is not necessary for survival or even proliferation of U2OS cells.

### KDM6A addiction is KDM6B-independent

**Fig 3:**
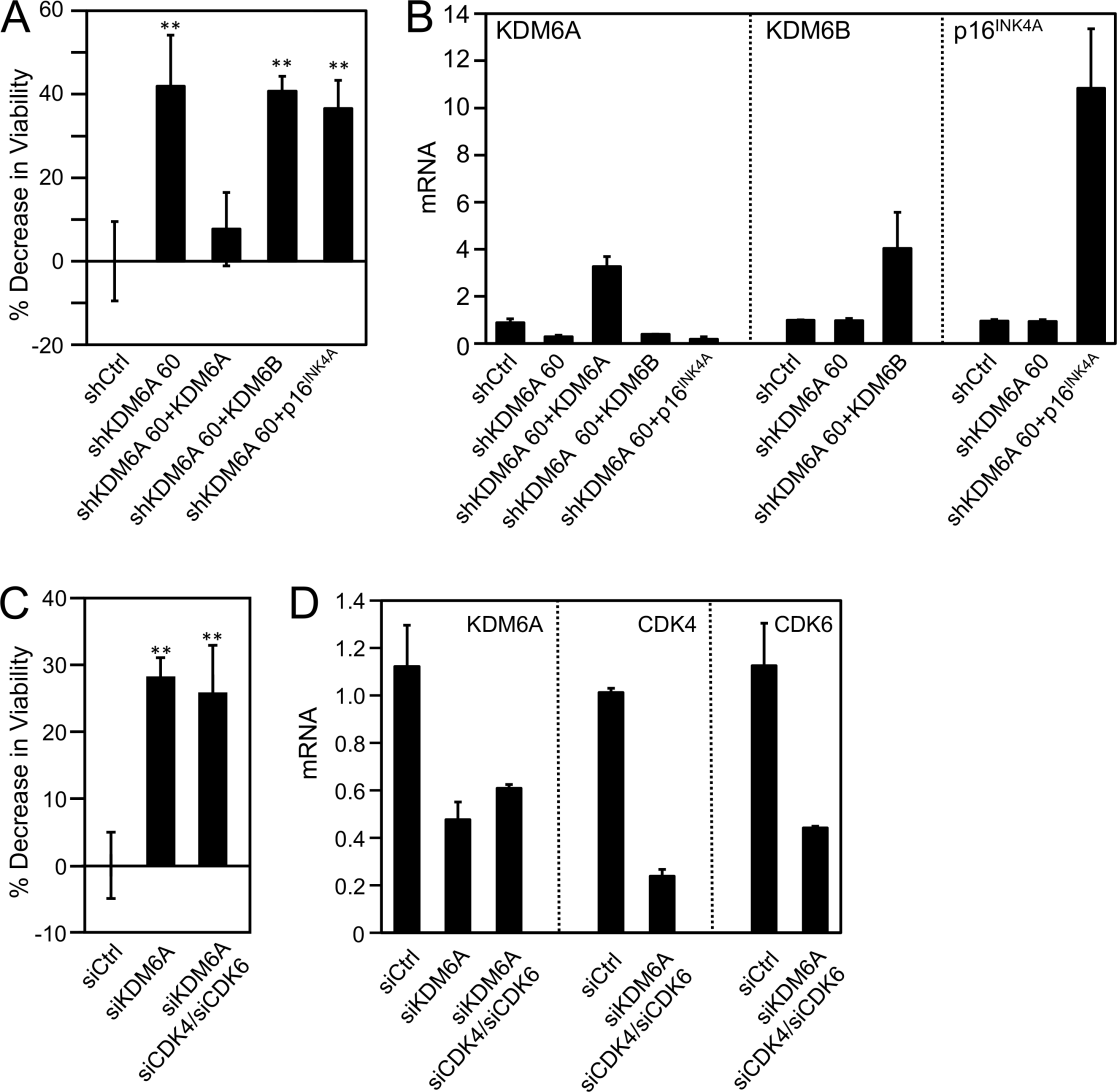
KDM6A addiction is KDM6B-independent. *(A*, *B)* KDM6A was depleted, and KDM6A, KDM6B, or p16^INK4A^ was expressed in the HPV16 positive CaSki cervical cancer line; *(A)* Cell viability was measured by reduction of resazurin; *(B)* Depletion and expression were verified by quantitative real-time RT-PCR; *(C*,*D)* CDK4 and CDK6 were depleted by transfection with CDK4- and CDK6-specific siRNA duplexes in the HPV16 positive CaSki cervical carcinoma cell line; *(C)* Cell viability was measured by reduction of resazurin; *(D)* Depletion was verified by quantitative real-time RT-PCR, Averages and SDs for three independent experiments are shown. Statistically significant changes are indicated, **P < 0.01.

High-risk HPV E7 also induces expression of the related histone demethylase KDM6B and triggers KDM6B addiction [14, 15]. Since both of these enzymes erase H3K27me3 marks, we determined whether KDM6A and KDM6B addiction was mediated through the same downstream pathway and/or if it involved similar mediators. To address this issue, we first investigated whether ectopic KDM6B expression may rescue the loss of viability caused by KDM6A depletion in CaSki cervical cancer cells. Ectopic expression of KDM6B, which was verified by qRT-PCR (Fig 3B), did not inhibit the loss of viability upon KDM6A depletion (*P* = 0.1968, SiHa), while ectopic expression of a non-targetable KDM6A rescued the effects of KDM6A depletion (Fig 3A and 3B). Similarly, ectopic expression of the downstream effector of KDM6B-addiction, p16^INK4A^ (CDKN2A), in these cells did not rescue the loss of cell viability caused by KDM6A depletion (*P* = 0.1415) (Fig 3A and 3B). CDK4/6 depletion also did not override the effect of KDM6A depletion (*P =* 0.325) (Fig 3C and 3D). These results show that KDM6A addiction is mediated by different downstream targets than those that mediate KDM6B addiction and specifically that KDM6A addiction is not related to p16^INK4A^ and CDK4 and/or CDK6 inhibition.

### Cervical cancer cells are addicted to p21^CIP1^

Despite the fact that p16^INK4A^ and related CDK4/CDK6 inhibitors do not mediate KDM6A addiction, KDM6A depletion in high-risk HPV E7 expressing cells caused a similarly strong loss of cell viability as KDM6B depletion. Hence, we assessed whether cell cycle inhibitors other than p16^INK4A^ may be involved. It has been reported that KDM6A can modulate expression of the CDK2 inhibitor, p21^CIP1^ (CDKN1A) [39]. Interestingly, p21^CIP1^ levels are high in HPV E7 expressing cells, and E7 has been reported to dampen its inhibitory activities [40, 41].

**Fig 4:**
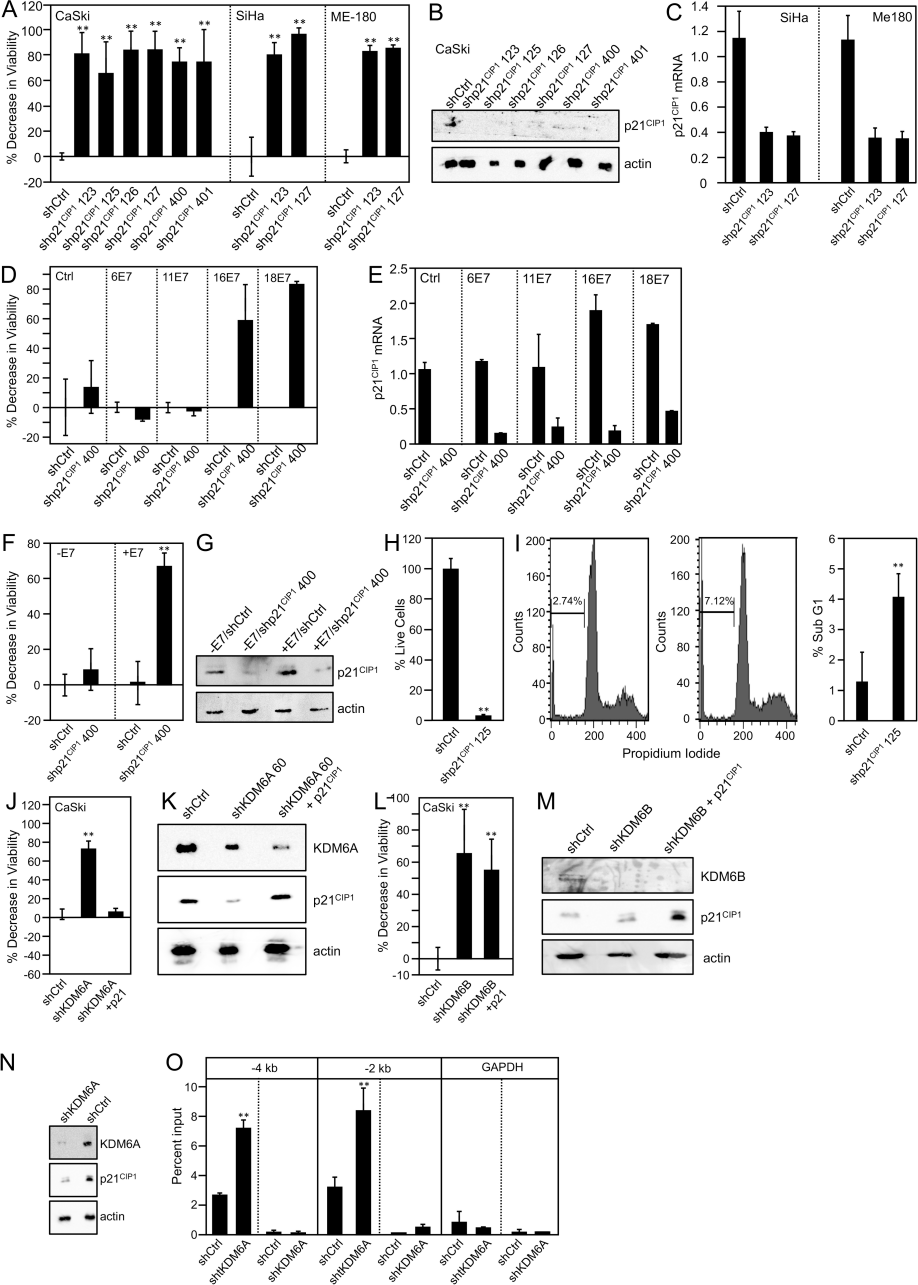
KDM6A addiction is mediated by p21^CIP1^. *(A-C)* p21^CIP1^ was depleted in the HPV16 positive CaSki and SiHa cervical carcinoma cell lines, and the HPV39 positive Me-180 cervical cancer line. Six independent p21^CIP1^ shRNA constructs (123, 125, 126, 127, 400, and 401) were used in the initial experiments in the CaSki cell line and two independent p21^CIP1^ shRNA expression vectors (123 and 127) were used in the SiHa and Me-180 cell lines; *(A)* Cell viability was measured by reduction of resazurin; *(B)* p21^CIP1^ depletion in the CaSki cell line was verified by Western blot. Lysates were separated by SDS/PAGE, transferred, and probed for p21^CIP1^. An actin blot is included as a loading control; *(C)* p21^CIP1^ depletion in the SiHa and Me-180 cell lines was verified by quantitative real-time RT-PCR; *(D*,*E)* p21^CIP1^ was depleted in HFKs expressing HPV6, HPV11, HPV16, and HPV18 E7; *(D)* Cell viability was measured by reduction of resazurin; *(E)* p21^CIP1^ depletion was verified by quantitative real-time RT-PCR; *(F*,*G)* p21^CIP1^ was depleted in U2OS-tet on cells with doxycycline-inducible expression of HPV16 E7; *(F)* Cell viability was measured by reduction of resazurin; *(G)* p21^CIP1^ depletion was verified by Western blot. Lysates were separated by SDS/PAGE, transferred, and probed for p21^CIP1^. An actin blot is included as a loading control; *(H)* p21^CIP1^ was depleted in the HPV16 positive SiHa cervical carcinoma cell line. After 10 days of puromycin selection, live cells were stained with sulforhodamine B. The bar graph depicts the percentages of live cells averaged from three experiments; *(I)* Cell cycle profile determined by fluorescence-activated sorting from a representative experiment and percentage of sub-G1 phase from three independent experiments; *(J*,*K)* KDM6A was depleted and p21^CIP1^ was expressed in the HPV16 positive CaSki cervical carcinoma cell line; *(J)* Cell viability was measured by reduction of resazurin; *(K)* Depletion and expression were verified by immunoblotting. Lysates were separated by SDS/PAGE, transferred, and probed for KDM6A and p21^CIP1^. An actin blot is included as a loading control; *(L*,*M)* KDM6B was depleted and p21^CIP1^ was expressed in the HPV16 positive CaSki cervical carcinoma cell line; (*L)* Cell viability was measured by reduction of resazurin; *(M)* Depletion and expression were verified by immunoblotting. Lysates were separated by SDS/PAGE, transferred, and probed for KDM6B and p21^CIP1^. An actin blot is included as a loading control; *(N)* KDM6A was depleted in the HPV16 positive CaSki cervical carcinoma cells and p21^CIP1^ expression was analyzed by Western blotting at 72 h post-transfection. An actin blot is shown as a loading control. *(O)* ChIP assays using lysates from SiHa cells using an antibody specific for H3K27me3 or the negative control IgG. qPCR was used to measure the degree of enrichment, and the results for each primer pair that captures sites at -4 kB and -2 kB in the p21^CIP1^ promoter are presented as a percentage of bound/input. Statistically significant changes (*P* < 0.05) are indicated by an asterisk. Fold enrichment over IgG was determined and is shown for each primer pair that capture sites at -4 kB and -2 kB in the p21^CIP1^ promoter. Averages and SDs for three independent experiments are shown. Statistically significant changes are indicated, **P < 0.01.

If p21^CIP1^ was key to KDM6A addiction, HPV E7 expressing cells would also be addicted to p21^CIP1^ expression. To test this hypothesis, we depleted p21^CIP1^ in the HPV16 positive SiHa and CaSki and the HPV39 positive Me-180 cervical carcinoma lines, and assessed cell viability. Depletion of p21^CIP1^ depletion with multiple different shRNAs was verified by immunoblotting and qRT-PCR (Fig 4B and 4C) and significantly decreased viability of CaSki (ranging from 66%; *P* = 0.0003 to 84%; *P* < 0.0001), SiHa (ranging from 81%; *P* < 0.0001 to 96%; *P* < 0.0001), and Me-180 cells (ranging from 83%; *P* < 0.0001 to 86%; *P* < 0.0001 (Fig 4A). These results revealed that HPV positive cervical cancer cells are addicted to p21^CIP1^ expression and suggest that p21^CIP1^ mediates KDM6A addiction.

Since high-risk HPV E7 oncoprotein expression was sufficient to generate KDM6A addiction, we hypothesized that high-risk HPV E7 expression may also be sufficient to generate p21^CIP1^ addiction. To test this hypothesis, we utilized primary HFK populations with ectopic expression of the high-risk HPV16 or HPV18 E7 or the low-risk HPV6 or HPV11 E7 proteins (Fig 2D and 2H). p21^CIP1^ was depleted by infection with lentiviral shRNA expression vectors, depletion was verified by qRT-PCR (Fig 4E), and cell viability was assessed. We observed significant 58.8% (*P* = 0.0199) and 83.2% (*P* <0.0001) decreases in viability upon p21^CIP1^ depletion in HPV16 E7 and HPV18 E7-expressing HFKs, respectively. In contrast, low-risk HPV6 and 11 E7 expressing HFKs were not significantly affected (P = 0.4126 and 0.2908, respectively) (Fig 4D). These results show that high-risk HPV E7 expression is sufficient to cause p21^CIP1^ addiction.

To determine if p21^CIP1^ addiction arises as a direct and immediate consequence of HPV16 E7 expression, we depleted p21^CIP1^ in osteosarcoma cells with doxycycline-inducible expression of HPV16 E7. Depletion of p21^CIP1^ (Fig 4G) did not significantly inhibit the viability of these cells before HPV16 E7 induction (*P* = 0.1346). In contrast, p21^CIP1^ depletion caused a significant 67% (*P* < 0.0001) decrease in viability after HPV16 E7 expression was induced by 72 h of doxycycline treatment which was rescued by ectopic expression of a non-targetable p21^CIP1^ (Fig 4F).

Ectopic p21^CIP1^ expression rescued the decrease in viability of KDM6A depleted CaSki cervical cancer cells *(P* < 0.0001, KDM6A depletion compared to KDM6A depletion plus p21^CIP1^ expression) and HPV16 E7 expressing HFKs *(P* < 0.0001, KDM6A depletion compared to KDM6A depletion plus p21^CIP1^ expression) (Fig 4J and 4K). In contrast however, ectopic p21^CIP1^ expression did not rescue inhibition of viability of KDM6B depleted CaSki cervical cancer cells (*P* = 0.472 KDM6B depletion compared to KDM6B depletion plus p21^CIP1^ expression) (Fig 4L and 4M). Moreover, KDM6A depletion in CaSki and SiHa cells resulted in decreased p21^CIP1^ protein expression (Fig 4N) and caused a decrease of the repressive H3K27me3 mark at the CDKN1A promoter (Fig 4O), functionally linking KDM6A and p21^CIP1^. In addition to cell viability assays, we also determined cell numbers by SRB assays following infection of CaSki cervical carcinoma cells with the recombinant p21^CIP1^ shRNA 125 expressing lentivirus at ten days after puromycin selection (Fig 4H). These experiments revealed that cell numbers were significantly decreased by 96.43% (*P* = 0.02). Moreover, FACS analysis of SiHa cervical cancer cells showed a significant 7.12% (*P* = 0.0012) increase in cells with a sub G0/G1 DNA content, supporting the notion that KDM6A depletion in SiHa cells causes apoptotic cell death (Fig 4I).

In summary, these results show that high-risk HPV E7 expressing cells are addicted to p21^CIP1^ expression and that p21^CIP1^ is a major mediator of KDM6A addiction.

### Addiction of E7 expressing cells to p21 ^CIP1^ is dependent on the integrity of the PCNA-interacting protein (PIP) box

**Fig 5:**
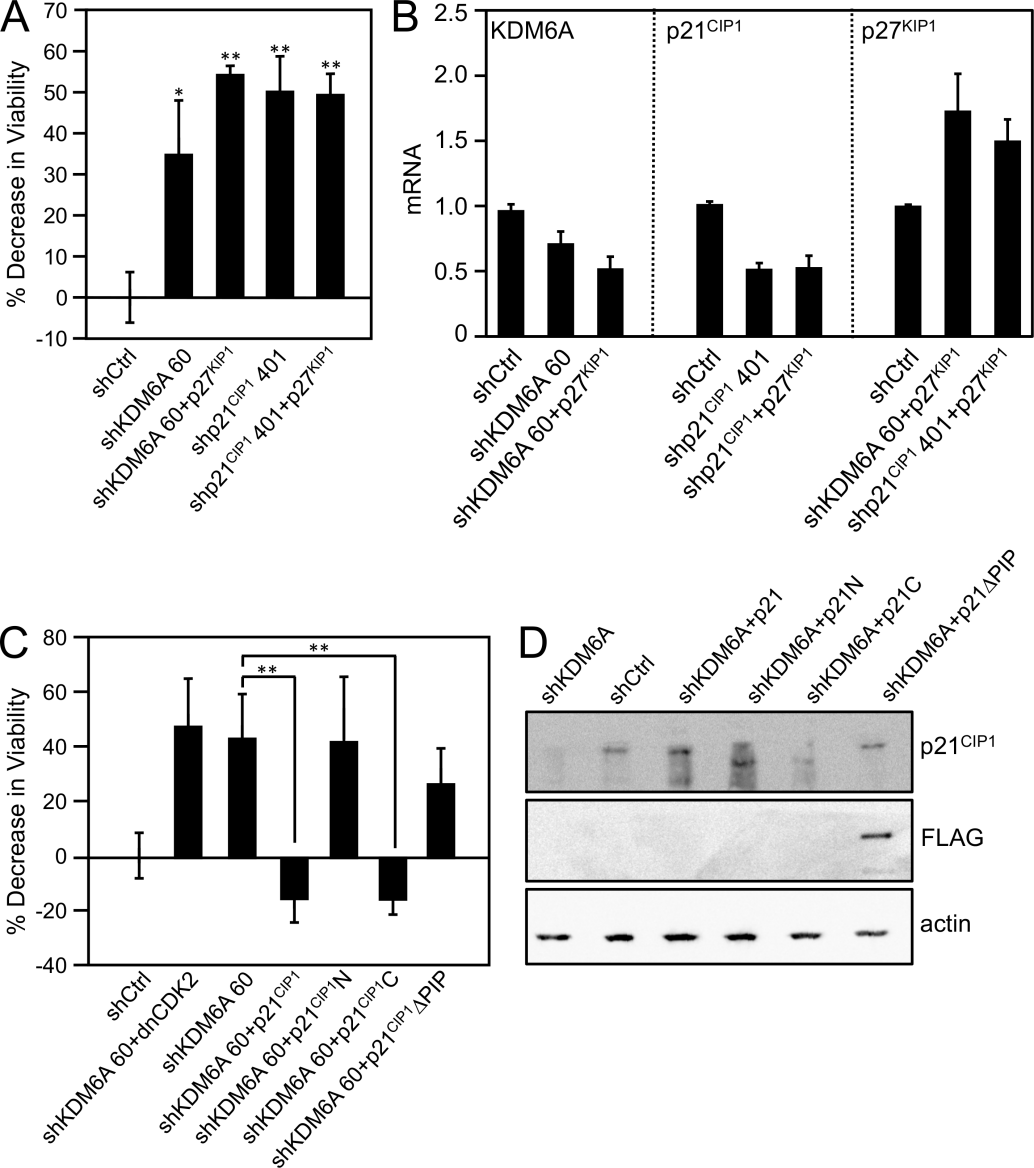
Rescue of KDM6A addiction by p21^CIP1^ is dependent on the integrity of the PCNA binding site. *(A*,*B)* Ectopic expression of the CDK2 inhibitor p27^KIP1^, which lacks PCNA interaction, does not rescue loss of KDM6A or p21^CIP1^ KDM6A or p21^CIP1^ were depleted in the HPV16-positive CaSki cervical carcinoma cell line and p27 was expressed; *(A)* Cell viability was measured by reduction of resazurin; *(B)* Depletion and expression were verified by quantitative real-time RT-PCR; *(C*,*D)* Ectopic expression of the C-terminal PCNA domain of p21^CIP1^ rescues KDM6A addiction, whereas expression of the N-terminal CDK2 inhibitor domain of p21^CIP1^, a PCNA binding defective p21^CIP1^ mutant, or a dominant negative mutant fails to rescue; *C)* Cell viability was measured by reduction of resazurin; *(D)* Expression was verified by immunoblot. Lysates were separated by SDS/PAGE, transferred, and probed p21^CIP1^. An actin blot is included as a loading control. Experiments were performed in CaSki cells, bars indicate averages and SDs of three experiments. Statistically significant changes are indicated, **P < 0.01.

The cyclin-dependent kinase (CDK) inhibitor p21^CIP1^ has amino-terminal cyclin and CDK-binding motifs [42–44] and a carboxyl-terminal PIP box that mediates binding to the proliferating cell nuclear antigen (PCNA) [45, 46]. Despite the fact that both p21^CIP1^ is expressed at high levels in HPV-positive cells, HPV E7 interferes with the ability of p21^CIP1^ to inhibit CDK2 activity and PCNA-dependent DNA replication [40, 41, 47]. If p21^CIP1^ addiction of HPV E7 expressing cells were related to CDK2 inhibition, ectopic expression of p27^KIP1^ (CDKN1B), which has similar CDK2 binding site and CDK2 inhibitory activity as p21^CIP1^ but does not inhibit replication through PCNA binding, would be predicted to rescue the loss cell viability caused by depletion of either KDM6A or p21^CIP1^. However, this was not observed (Fig 5A and 5B). Similarly, ectopic expression of a dominant negative CDK2 mutant [48] did not rescue the loss of cell viability caused by KDM6A depletion (Fig 5C and 5D). Moreover, ectopic expression of the amino-terminal p21^CIP1^ fragment that contains the CDK2 binding domain and is sufficient for CDK2 inhibition [42] did not rescue the loss of cell viability in response to KDM6A depletion (Fig 5C). Collectively, these results show that p21^CIP1^ addiction of high-risk HPV E7 expressing cells is not related to CDK2 inhibition.

In contrast, ectopic expression of the carboxyl terminal p21^CIP1^ domain, which contains the PCNA binding site and is sufficient to inhibit PCNA [42], efficiently rescued the decrease in viability upon KDM6A depletion. Moreover, expression of p21^CIP1^ carrying a mutation in the PCNA-interacting protein (PIP) box that disrupts PCNA binding and inhibition [49] did not rescue the decrease in cell viability upon KDM6A depletion (Fig 5C). Collectively, these results show KDM6A and p21^CIP1^ addiction of high-risk HPV E7 expressing is related to PCNA binding and independent of CDK2 inhibition.

### Depletion of replication factors rescue KDM6A and p21^CIP1^ addiction

**Fig 6:**
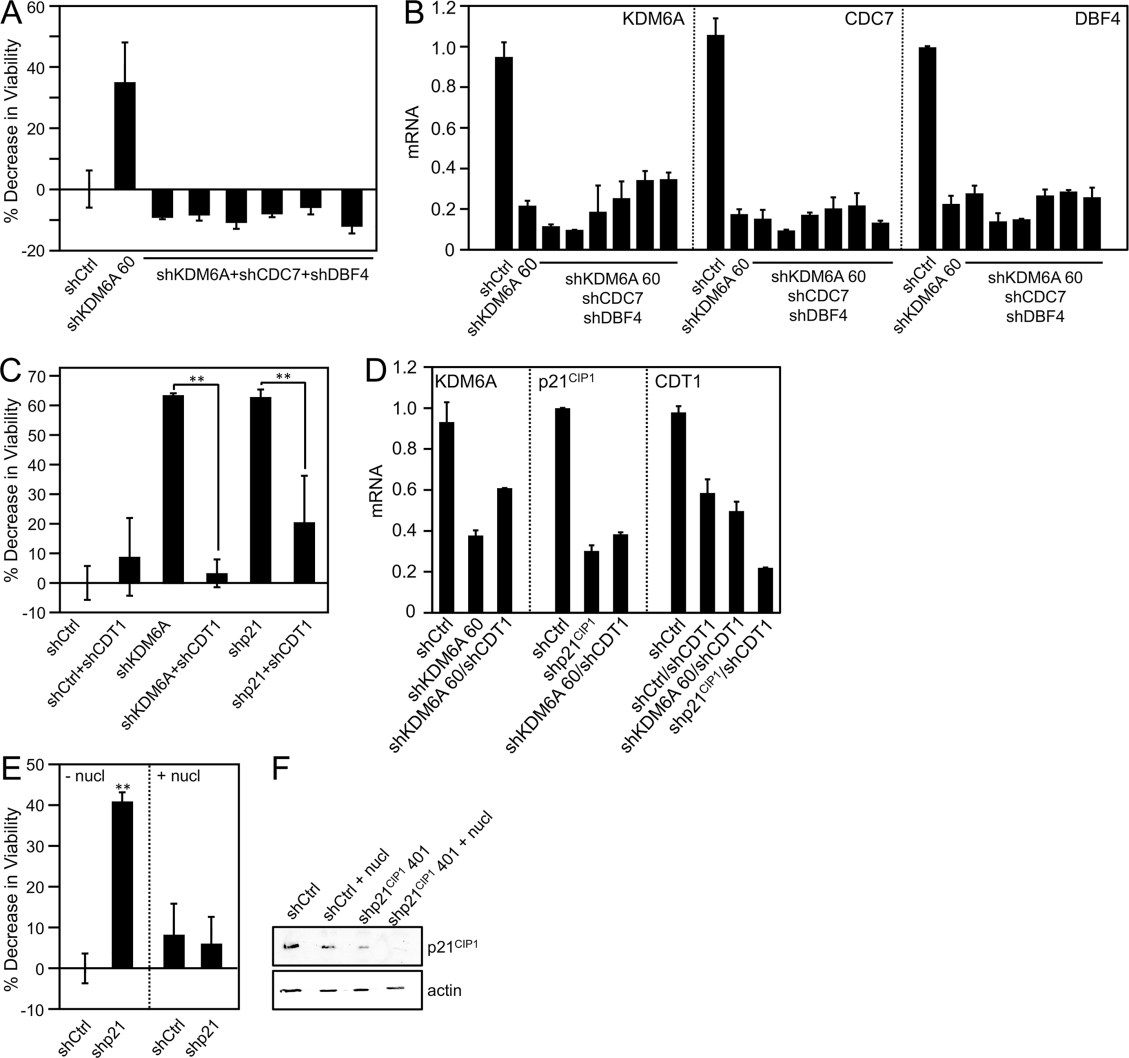
Decrease in viability in response to KDM6A/p21^CIP1^ depletion in E7 expressing cells may be a consequence of replication stress. *(A*,*B)* Depletion of the CDC7/DBF4 kinase rescues KDM6A loss. Each shKDM6A + shCDC7 + shDBF4 depletion contained shKDM6A 60 and the following combinations of shCDC7 and shDBF4, in order: shCDC7 168/shDBF4 954, shCDC7 169/shDBF4 955, shCDC7 170/shDBF4 956, shCDC7 171/shDBF4 957, shCDC7 169/shDBF4 954, and shCDC7 172/shDBF4 957; *(A)* Cell viability was measured by reduction of resazurin; *(B)* Depletion was verified by quantitative real-time RT-PCR; *(C*,*D)* CDT1 depletion rescues KDM6A and p21^CIP1^ loss; *(C)* Cell viability was measured by reduction of resazurin; *(B)* Depletion was verified by quantitative real-time RT-PCR; *(E*,*F)* Supplementing E7 expressing CaSki cells with exogenous nucleosides inhibits cell death in response to p21^CIP1^ depletion. p21^CIP1^ was depleted in U2OS osteosarcoma cells with doxycycline-inducible HPV16 E7 expression and supplemented with 50 μM nucleosides in the media or grown under standard conditions; *(E)* Cell viability was measured by reduction of resazurin; *(F)* Depletion was verified by immunoblot. Lysates were separated by SDS/PAGE, transferred, and probed p21^CIP1^. An actin blot is included as a loading control. Bar graphs represent averages and SDs of three experiments, statistically significant changes are indicated, **P < 0.01.

We next attempted to directly assess the necessity of PCNA for p21^CIP1^ addiction. However, treatment of cells with T2 amino alcohol (T2AA), a compound that disrupts interaction of PCNA with PIP box binding proteins, caused extensive cell death even in normal cells. This is not surprising, given that PCNA has multiple functions in DNA replication and DNA repair [50]. To nevertheless address whether the ability of p21^CIP1^ to inhibit replication is key to addiction of high-risk HPV E7 expressing cells to KDM6A, we assessed whether depleting the DNA licensing factors CDC7/DBF4 in SiHa cervical cancer cells might abrogate the KDM6A-mediated decrease in cell viability. The CDC7/DBF4 complex acts as a protein kinase that is required for the initiation of DNA replication [51, 52]. Depletion of CDC7/DBF4 significantly inhibited the decrease of viability in response to loss of KDM6A expression. (Fig 6A and 6B). Similarly, depletion of the pre-replication complex component, CDT1, rescued the loss of viability caused by p21^CIP1^ depletion (Fig 6C and 6D). These results support our hypothesis that the loss of viability of high-risk HPV expressing cells caused by KDM6A and p21^CIP1^ depletion are mechanistically connected to induction of aberrant cellular DNA replication.

### Exogenous nucleoside supplementation rescues p21^CIP1^ addiction

Aberrant firing of replication origins results in replication stress, a frequent hallmark of cancer cells. Replication stress triggers double strand DNA breaks and causes activation of the ATM and ATR kinases. These in turn causes genomic instability, and in severe cases when the DNA damage cannot be repaired, cell death. ATR is hyperactive in HPV E7 expressing cells [53–56], and HPV16 E7 is known to induce replication stress due to aberrant E2F activity as a consequence of pRB inactivation and through other mechanisms [57]. Uncontrolled firing of replication origins can cause nucleotide starvation, and exogenous nucleoside supplementation has been shown to attenuate the replication stress response [58–60]. We hypothesized that high-level p21^CIP1^ expression in E7 expressing cells may dampen replication stress, presumably by complexing PCNA. Therefore, we tested whether dampening replication stress by nucleoside supplementation might rescue the loss of cell viability in response to p21^CIP1^ depletion. U2OS osteosarcoma cells with doxycycline-inducible HPV16 E7 expression were supplemented with 50 μM nucleosides in the media or grown under standard conditions. In contrast to cells grown under standard conditions, p21^CIP1^ depletion in U2OS osteosarcoma cells with doxycycline-inducible HPV16 E7 expression did not cause a significant loss of viability in nucleoside-supplemented cells (Fig 6E and 6F). These results are consistent with a model whereby KDM6A and p21^CIP1^ addiction is based on p21^CIP1^ dampening replication stress of high-risk HPV E7 expressing cells.

### Depletion of KDM6A and p21^CIP1^ causes an increase in 53BP1 nuclear bodies

**Fig 7:**
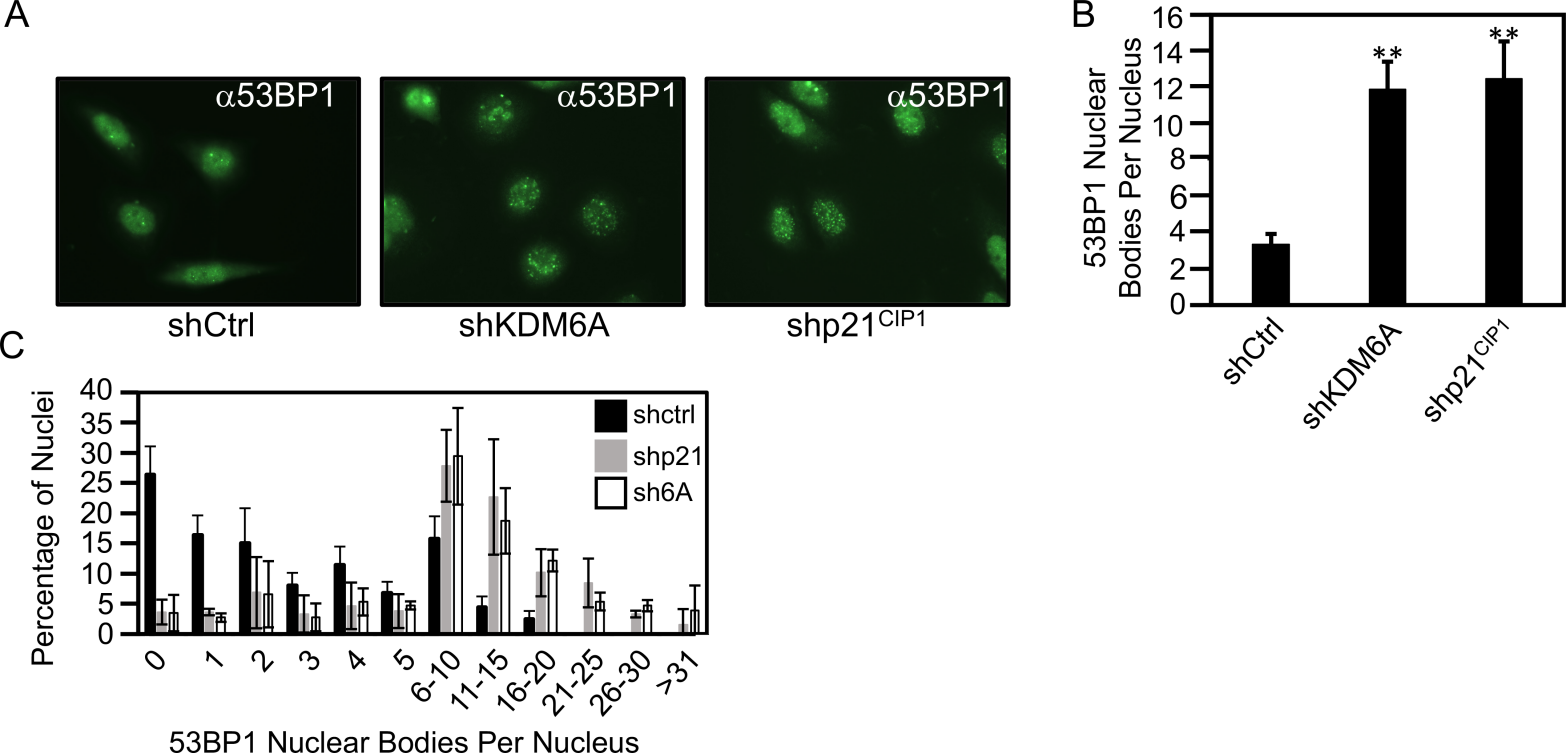
KDM6A/p21^CIP1^ depletion in E7 expressing cells causes 53BP1 nuclear foci formation. KDM6A or p21^CIP1^ were depleted in the HPV16 positive SiHa cervical carcinoma cells. *(A)* The cells were immunostained with an antibody against 53BP1; *(B)* 53BP1 nuclear foci in 100 cells per experiment were quantified; and *(C)* Quantification of 53BP1 nuclear foci per nucleus upon KDM6A/p21^CIP1^ depletion. Bar graphs represent averages and SDs of six experiments, statistically significant changes are indicated, **P < 0.01.

Replication stress causes single and double strand DNA breaks that are sensed by the ATR and ATM kinases. 53BP1 nuclear bodies are markers of DNA breaks, including those induced by replication stress. [61]. To determine whether KDM6A and/or p21^CIP1^ induction by E7 affected the incidence of DNA double strand breaks, we evaluated the number of 53BP1 nuclear bodies in KDM6A and p21^CIP1^ depleted SiHa cervical cancer cells. Depletion of KDM6A or p21^CIP1^ caused a 3.5 fold (P = 0.0177) or 3.7 fold (P = 0.0172) increase in 53BP1 nuclear bodies, respectively (Fig 7). These results further support the hypothesis that KDM6A and p21^CIP1^ addiction is based on the role of p21^CIP1^ in dampening replication stress in E7 expressing cells.

### Depletion of KDM6A and p21^CIP1^ causes an increase in re-replication

**Fig 8:**
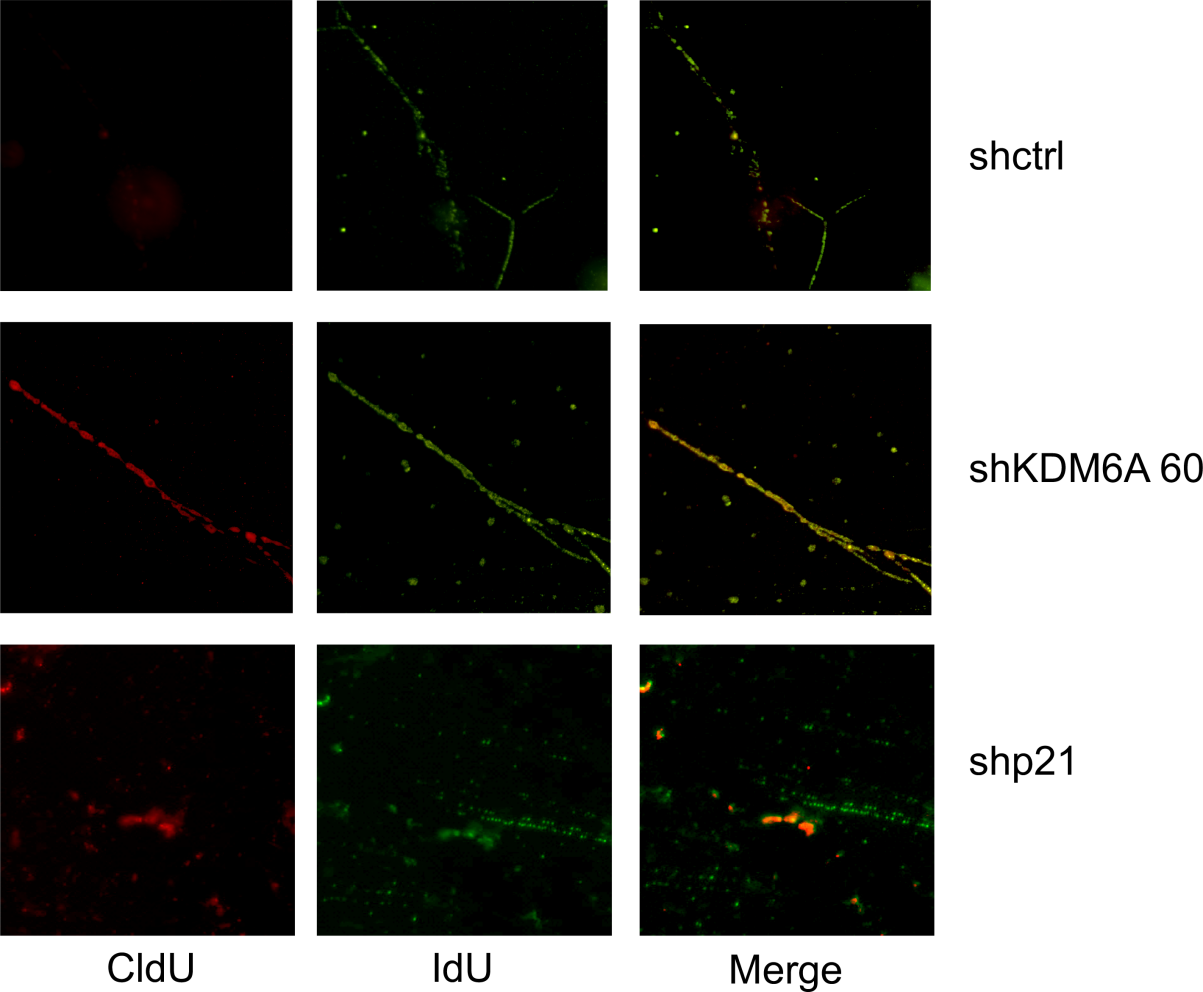
KDM6A/p21^CIP1^ depletion in E7 expressing cells causes re-replication. KDM6A or p21^CIP1^ were depleted in the HPV16 positive SiHa cervical carcinoma cells. Example of single combed DNA molecules labeled with IdU (green) and CldU (red).

To further analyze the hypothesis that KDM6A and p21^CIP1^ addiction is based on the role of p21^CIP1^ in dampening replication stress in E7 expressing cells, we analyzed replication in individual DNA fibers in KDM6A and p21^CIP1^ depleted SiHa cervical cancer cells. Depletion of KDM6A or p21^CIP1^ caused an increase in the amount of re-replication, as detected by yellow tracks (average Pearson correlation coefficients = 0.16 (control cells), 0.48 (KDM6A-depleted cells), and 0.34 (p21^CIP1^-depleted cells), adding further support to the hypothesis that KDM6A and p21^CIP1^ addiction is based on the role of p21^CIP1^ in dampening replication stress in E7 expressing cells (Fig 8).

## Discussion

Similar to oncogenic mutations in cellular oncogenes and tumor suppressors, infections with oncogenic viruses trigger innate tumor suppressor pathways. The evolution of these viruses, however, has been driven by the need to overcome such cellular defense responses. Similar to what has been reported for the MYC oncogene [62–65], high-risk HPV E7 oncogene expressing cells are predisposed to undergo cell death particularly under conditions of limited growth factor availability, but high-risk HPV E6 proteins that are co-expressed during a viral infection effectively abrogate this response [66, 67]. Similarly, we have shown that high-risk HPV E7 induces p16^INK4A^ expression, similar to what has been reported for RAS expression causes “Oncogene-Induced-Senescence” (OIS) which is mediated by epigenetic de-repression of p16^INK4A^ through the H3K27me3-specific histone demethylase KDM6B [14, 15, 35, 36]. To evade elimination by OIS, high-risk HPVs have evolved to target the key OIS mediator, RB1, for proteasomal degradation [68–71]. Most remarkably, HPV E7 expressing cells, including some cervical carcinoma cell lines become acutely “addicted” to KDM6B and p16^INK4A^ expression [14]. While “oncogene-addiction” is a well-known concept [38], addiction of cancer cells to expression of a tumor suppressor, such as p16^INK4A^, appears counterintuitive, at best.

**Fig 9:**
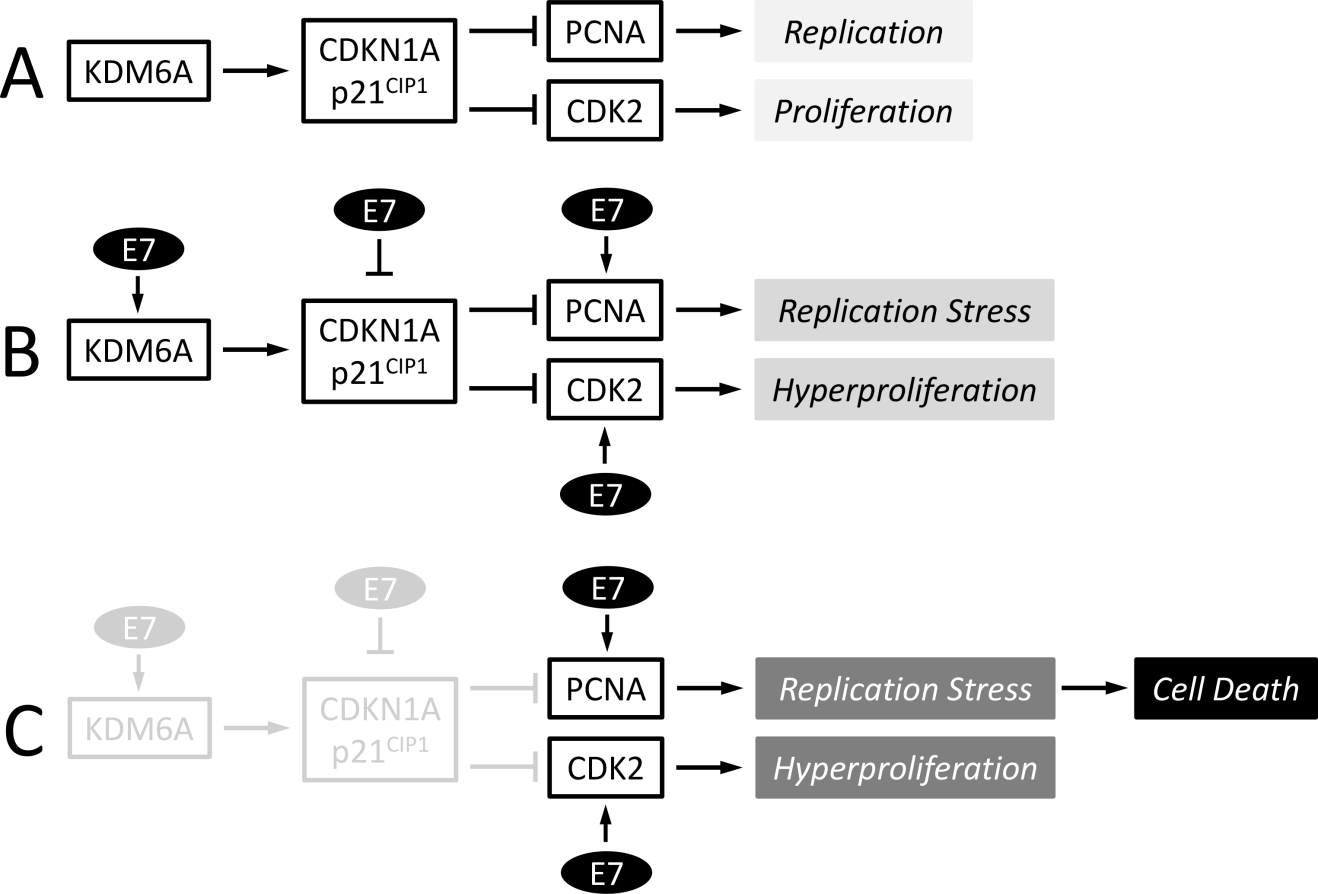
**The p21^CIP1^ regulatory circuit in normal (A) high-risk HPV E6/E7 expressing cells (B), and high-risk HPV E6/E7 expressing cells depleted of KDM6A and/or p21^CIP1^ (C).** See text for details.

HPV16 E7 expression also causes increased KDM6A expression and addiction to KDM6A. Like KDM6B, KDM6A erases H3K27me3 repressive marks, thereby counteracting polycomb repression [23, 26, 27]. Despite this similarity in enzymatic activities, the two enzymes are involved in de-repressing distinct cellular targets [23, 24, 26, 27, 72–76]. Here, we show that KDM6A addiction is mechanistically distinct from KDM6B and is mediated by the CDK2 and PCNA inhibitor, p21^CIP1^. It is likely that, similar to what has been reported for KDM6B, increased KDM6A expression also represents a cellular defense response to expression of high-risk HPV E7 proteins. KDM6A mediated epigenetic de-repression of p21^CIP1^ will inhibit cell cycle progression through CDK2 inhibition and DNA replication through binding and inhibiting PCNA. High-risk HPVs, however, have evolved to short-circuit this growth-inhibitory cellular defense response by dampening the cell cycle and replication inhibitory activities of p21^CIP1^ as well as through other mechanisms including enhanced expression of E2F regulated genes, many of which stimulate cell cycle progression and DNA replication (Fig 9). The ability of E7 to abrogate the CDK2 inhibition by p21^CIP1^ may be key to retaining differentiating keratinocytes in a replication competent state [77, 78], which is an essential requirement for HPV genome amplification and progeny synthesis in differentiated keratinocytes.

Our experiments, however, yielded no evidence that the CDK2 inhibitory activity of p21^CIP1^ was rate limiting for KDM6A addiction. Specifically, ectopic expression of p27^KIP1^, which contains a structurally related CDK2 interacting/inhibitory domain, or a kinase defective, dominant negative CDK2 mutant did not rescue the antiproliferative effect of KDM6A depletion. Rather, we discovered that the ability of p21^CIP1^ to bind and inhibit PCNA was necessary. PCNA is an E2F-responsive gene that is highly expressed in HPV E7 expressing cells and forms a trimeric “sliding clamp” that enhances the interaction of the DNA polymerase with the template DNA, thereby enhancing the processivity of the replicative DNA polymerase and coordinating DNA damage repair during DNA replication (reviewed in [79, 80]). In addition, post-translationally modified versions of PCNA also play important roles in post-replicative DNA damage repair. The p21^CIP1^ protein binds to PCNA through a PIP box, a conserved motif that is present in various PCNA interacting proteins that are involved in DNA replication. E7, in turn, has been reported to bind p21^CIP1^ thereby abrogating the PCNA inhibitory activity of p21^CIP1^[41]. Given that p21^CIP1^ is highly expressed in E7 expressing cells [81–83], and that p21^CIP1^ inhibition by E7 is based on a stoichiometric interaction [40, 41], it is likely that there is a significant pool of “active”, PCNA inhibitory p21^CIP1^ in E7 expressing cells. Our findings that (a) the PIP box in p21^CIP1^ is necessary to rescue the loss of cell viability caused by KDM6A depletion and (b) that the loss of cell viability caused by KDM6A or p21^CIP1^ depletion is abrogated by simultaneous depletion of replication factors are consistent with the model that high-risk HPV E7 expressing cells are addicted to PCNA inhibition by p21^CIP1^.

Why, though, would it be essential to inhibit PCNA in high-risk HPV E7 expressing cells when the major challenge of the viral life cycle is to keep the cellular replication machinery active for viral genome amplification and progeny synthesis? Expression of replication regulatory proteins is a highly choreographed process that is tightly linked to the cell division cycle. Expression of many of these factors, including PCNA, is modulated by members of the E2F family of transcription factors. HPV E7 is well known to subvert E2F regulation by binding and enhancing the degradation of the RB1 tumor suppressor and the related RBL1 (p107) and RBL2 (p130) proteins as well as through other mechanisms [16, 68–71, 84]. Therefore, E2F-regulated replication factors, including PCNA, are highly expressed in E7 expressing cells. Aberrant firing of replication origins can lead to a process referred to as “replication stress”, which arises when replication forks stall. Single stranded DNA within these structures will trigger activation of the ataxia telangiectasia and Rad3 related kinase (ATR). ATR activation is critical to resolve replication stress and to limit re-replication by engaging an S-phase checkpoint that is mediated by the TP53 as well as the RB1 tumor suppressor pathways [85, 86]. In cells that have suffered TP53 and/or RB1 mutations, replication stress can generate genomic instability. Replication stress is a frequent hallmark of human tumors, and HPV16 E7 expression in normal diploid human cells induces hallmarks of replication stress [57, 59] and double strand DNA breaks [87]. Most remarkable, E7-induced genomic instability can be ameliorated by supplementation with exogenous nucleosides, suggesting that E7 induced double strand DNA breaks and the ensuing genomic instability is at least in part triggered by replication stress [88]. E7-induced replication stress in differentiated epithelial cells, however, may be beneficial to the viral life cycle since activation of the double strand DNA break response, including ATM and ATR, enhance viral genome maintenance and amplification [53–55, 89–91].

Our results suggest a model whereby p21^CIP1^ and specifically its ability to inhibit PCNA is necessary to limit high-risk HPV E7 induced replication stress to levels that cells can tolerate and do not markedly interfere with viability. Depletion of p21^CIP1^ in E7 expressing cells, however, enhanced replication stress and the associated double strand DNA breaks as evidenced by 53BP1 nuclear foci and the resulting of cell viability. The fact that nucleoside supplementation, which alleviates E7-mediated replication stress, can effectively dampen the observed loss of viability in response to p21^CIP1^ depletion strongly supports this model.

Our results also suggest a model whereby KDM6A expression by an oncogenic insult caused by E7 expression. The cell cycle and replication inhibitor p21^CIP1^ is a rate-limiting component of this KDM6A-mediated cellular defense response that removes a potentially pre-carcinogenic cell from the proliferative pool by shutting down S-phase entry and DNA replication. High-risk HPVs, however, have evolved to evade this response by dampening the CDK2 as well as PCNA inhibitory activities of p21^CIP1^ through multiple mechanisms. Consequently, despite high-level p21^CIP1^ expression, high-risk HPV E7 expressing cells retain CDK2 activity and remain proliferatively active, but this generates some replication stress. E7-triggered replication stress in differentiating epithelial cells is not only an acceptable price to pay for the resulting abundant availability of cellular replication factors, but HPVs have evolved to take advantage of the resulting ATR/ATM activation for the replication of their genomes [53, 89]. Nonetheless, high-level p21^CIP1^ expression is essential for viability of high-risk HPV E7 expressing cells since it keeps replication stress at a manageable level; loss of KDM6A or p21^CIP1^ causes cell death that is at least in part due to replication stress. Since many cervical carcinoma lines remain KDM6A and p21^CIP1^ addicted, this suggests that this pathway may be targetable for therapeutic intervention.

In summary, our results show that KDM6A induction represents a cellular defense response to HPV E7 oncogene expression that is mechanistically independent and different from the KDM6B mediated response that we discovered previously [14]. KDM6B triggers p16^INK4A^ expression causing activation of the RB tumor suppressor pathway which signals cellular senescence, whereas KDM6A activates p21^CIP1^ expression which corresponds to the cell cycle and replication inhibitory arms of the p53 tumor suppressor pathway. In each case, the virus had to adapt and evolve strategies to overcome these abortive cellular responses. In the case of KDM6B, high-risk HPV E7 proteins cause RB1 destabilization, thereby short-circuiting the RB1-mediated senescence response. In the case of KDM6A, high-risk HPV E7 proteins inhibit the cell cycle and replication inhibitory activities of p21^CIP1^. This causes replication stress and DNA breaks, which activates the DNA repair machinery that these viruses harness for their own genome replication. Dysregulated cell cycle entry as a consequence of RB1 loss and CDK2 hyperactivity causes hyperproliferation that, in concert with the increased incidence of double strand DNA breaks, can cause genomic instability and malignant progression. The HPV-mediated subversion of the tumor suppressive activities of KDM6A and KDM6B and the ensuing addiction to these two enzymes of high-risk HPV expressing cells, but not normal cells, is not only of academic interest, but also provides novel therapeutic targets for high-risk HPV-associated lesions and cancers.

## Materials and methods

### Cells

Primary HFKs were isolated and cultured as previously described [16]. HFKs were transduced by recombinant retroviruses carrying either the control vector (LXSN) or vectors encoding HPV16 E6, HPV16 E7, HPV16E6 and E7, or HPV18 E7 [92]. HPV16 and 18 E6 and E7 expression was assessed by quantitative RT-PCR as previously described [14]. U2OS-tet on (Clontech), CaSki (ATCC), SiHa (ATCC), Me-180 (ATCC), and HeLa cells (ATCC) were maintained as previously described [14]. For experiments on the inhibition of PCNA interaction, cells were treated with T2AA (Sigma) for 72 h.

### Ethics statement

The human keratinocytes used in this study were obtained from discarded foreskin circumcisions from anonymous donors at Brigham and Women’s Hospital and are not classified as human subjects research. These specimens were not specifically collected for this study and lack all identifiers.

### Nucleoside supplementation

Uridine (Sigma) and cytidine (Sigma) were dissolved in distilled water to make 10 mM stocks, adenosine (Sigma) and guanosine (Sigma) were dissolved to make 2 mM stocks, and the suspensions was briefly boiled, filter sterilized, and added to complete medium at a final concentration of 50 μM.

### Plasmids, transfections, and lentiviral transduction

Transient transfections were performed using Polyethylenimine (PEI) (Polysciences) as described [93]. The cells were transfected with shRNA constructs and plasmids described in S1 and S2 Tables.

### Cell viability assays

Three days after transfection, media was removed, and 10 μg/ml resazurin sodium salt (Sigma; diluted in growth medium) was added to each well. The plates were incubated for 1–3 h at 37°C and then read at 570 and 600 nm on a microtiter well plate reader (Biotek). To assess longer term viability, cells were selected with 1 μg/mL of puromycin at 24 hours post infection. At 10 days post-selection, the surviving cells were stained with sulforhodamine B (Sigma), and quantified in a plate reader [94].

### Quantitative RT-PCR

Total RNA was extracted using the Quick-RNA MiniPrep Kit (Zymo), and cDNA was reverse transcribed using Taqman® Reverse Transcription Reagents (Life Technologies). Quantitative RT-PCR was performed using either Taqman qPCR assays for CDK4, CDK6, HPV16 E6, HPV6 E7, HPV11 E7, HPV16 E7, HPV18 E7, KDM6A, KDM6B, and p16^INK4A^ (supplied by Applied Biosystems as a 20× premix containing both primers and FAM-nonfluorescent quencher probe) or SYBR Green PCR Master Mix and the listed PCR primers (S3 Table) to analyze expression of CDT1, CDC7, DBF4, p21^CIP1^, and p27^KIP1^. Analysis was performed using a StepOnePlus Real-Time PCR System (Applied Biosystems). Data shown are calculated using the ΔΔCT method and are normalized to expression of 18s rRNA (Taqman) or GAPDH (SYBR Green) as the housekeeping gene.

### Chromatin immunoprecipitation

ChIP was performed using the Simple ChIP Plus Enzymatic Chromatin IP Kit (Cell Signaling). Immunoprecipitation of cross-linked chromatin was conducted the following antibodies: H3 (2560; Cell Signaling), H3K27me3 (ab6002; Abcam), and IgG (2729; Cell Signaling).

After immunoprecipitation, extracted DNA was amplified by real-time qPCR using the oligonucleotide primers described in S4 Table.

### Statistical methods

Student *t* test was used to evaluate statistical significance.

### Western blotting

Cell lysates were prepared and processed as described [16]. Antibodies were used at the following dilutions: β-actin (MAB1501, 1:1,000; Chemicon), KDM6A (ab36938, 3μg/mL; Abcam), p21^CIP1^ (ab109520, 1:750 and ab109199, 1:1,000; Abcam), FLAG (F-3165, 1:1,000; Sigma) and HRP-conjugated secondary anti-rabbit (1:10,000; Amersham) and anti-mouse (1:10,000; Amersham). Antigen/antibody complexes were visualized by enhanced chemiluminescence (PerkinElmer Life Sciences) and electronically acquired with a Kodak 4000R Image Station (Kodak) equipped with Carestream Molecular Imaging Software.

### Immunofluorescence

Immunofluorescence analysis of monolayer cells was performed as described [16] using anti-53BP1 (ab172580; Abcam) and secondary donkey anti-goat Alexa Fluor 488 secondary antibody (ab150077; Abcam). Nuclei were counterstained with Hoechst 33258. Images were acquired using an Axioplan 2 microscope (Zeiss) with a 63× objective and Axiovision 4.8 (Zeiss) software.

### Molecular combing assays

Unsynchronized cells were pulse labeled for 120 min with growth medium containing 10 mM of the thymidine analog iododeoxyuridine (IdU). At the end of the first labeling period, the cells were washed twice with warm medium and pulse labeled once more for 30 min with growth medium containing 10 mM of the thymidine analog chlorodeoxyuridine (CldU). Cells were then harvested, and genomic DNA was extracted and combed as previously described [95]. The primary antibody for fluorescence detection of IdU was mouse anti-BrdU (Becton Dickinson), and the secondary antibody was goat anti-mouse mouse-Dylight 488 (Abcam). The primary antibody for fluorescence detection of CldU was rat anti-CldU (Serotec). The secondary antibody was goat anti-rat Alexa Cy3 (Abcam).

## Supporting information

S1 Table List of shRNA constructs used in this study. (DOCX)

S2 Table List of plasmids used in this study. (DOCX)

S3 Table List of primers used for quantitative real-time RT PCR primers in this study. (DOCX)

S4 Table List of primers used for quantitative ChIP in this study. (DOCX)
