# Supplementary material for: KDM6A addiction of cervical carcinoma cell lines is triggered by E7 and mediated by p21CIP1 suppression of replication stress
Source: PLoS Pathog. 2017 Oct 2;13(10):e1006661. doi: 10.1371/journal.ppat.1006661 (PMC5638616; doi:10.1371/journal.ppat.1006661)
Supplement: S1 Table — (DOCX) [file ppat.1006661.s001.docx]

**Supplemental Table 1**

| shCDC7 | TRC Lentiviral Human CDC7 shRNA (TRCN0000003168, TRCN0000003169, TRCN0000003170, TRCN0000003171, TRCN0000003172; Dharmacon) |
| --- | --- |
| shCDT1 | TRC Lentiviral Human CDT1 shRNA (TRCN0000073298, TRCN0000073299, TRCN0000073301,TRCN0000073302; Dharmacon) |
| siCDK4 | cyclin-dependent kinases 4 (CDK4)-specific ON-TARGETplusSMARTpool (L-003238-00; Dharmacon) |
| siCDK6 | CDK6-specific ON-TARGETplusSMARTpool (L-003240-00; Dharmacon), |
| shDBF4 | TRC Lentiviral Human DBF4 shRNA  TRCN0000037954, TRCN0000037955, TRCN0000037956, TRCN0000037957, TRCN0000037958; Dharmacon) |
| shKDM6A | TRC Lentiviral Human KDM6A shRNA (TRCN0000107760, TRCN0000107761, TRCN0000107762; Dharmacon) |
| shKDM6B | TRC Lentiviral Human KDM6B shRNA (TRCN0000095268; Open Biosystems) |
| shp16^INK4A^ | pbabeshp16AB, pbabep16CD, and pbabe-  p16EF or pbabeU6 as a control (a generous gift from James Rocco, Massachusetts General Hospital, Boston, MA) |
| shp21^CIP1^ | TRC Lentiviral Human p21^CIP1^ shRNA (TRCN0000010123, TRCN0000010125, TRCN0000010126, TRCN0000010127, TRCN0000010400, TRCN0000010401; Dharmacon) |
| shRNA control vector | MISSION Non-Target shRNA control vector (Sigma) |
