# Supplementary material for: KDM6A addiction of cervical carcinoma cell lines is triggered by E7 and mediated by p21CIP1 suppression of replication stress
Source: PLoS Pathog. 2017 Oct 2;13(10):e1006661. doi: 10.1371/journal.ppat.1006661 (PMC5638616; doi:10.1371/journal.ppat.1006661)
Supplement: S2 Table — (DOCX) [file ppat.1006661.s002.docx]

**Supplemental Table 2**

| **CDK2-DN** | CDK2-DN AdCDK2-DN was a gift from Sander van den Heuvel (Addgene plasmid # 1882) [1] |
| --- | --- |
| **HPV6, 11, 16, and 18 E7** | MSCV-C, MSCV-C 6E7, MSCV-C 11E7, MSCV-C 16E7, MSCV-C 18E7 [2] |
| **KDM6A** | pCMV-HA-UTX was a gift from Kristian Helin (Addgene plasmid # 24168) [3] |
| KDM6B | MSCV JMJD3 was a gift from Paul Khavari (Addgene plasmid # 21212) [4] |
| **p16^INK4A^** | p16 Wild-type p16 INK4A(missing the first 24 nucleotides; a gift of Robert Weinberg, Whitehead Institute, Massachusetts Institute of Technology, Cambridge MA) [5] cloned into LXSN [6] |
| **p21** | pcDNA3.1 p21 a gift of Anindya Dutta (University of Virginia, Charlottesville, VA) [7] |
| **p21N** | pcDNA3.1 p21N a gift of Anindya Dutta (University of Virginia, Charlottesville, VA) [7] |
| **p21C** | pcDNA3.1 p21C a gift of Anindya Dutta (University of Virginia, Charlottesville, VA) [7] |
| **p21deltaPIP** | pEFFN p21deltaPIP a gift of Anindya Dutta (University of Virginia, Charlottesville, VA) [8] |
| **p27** | pcDNA3 p27 a gift of Joan Massagué ([Memorial Sloan Kettering Cancer Center](https://www.mskcc.org/research-areas/labs/joan-massague), New York, NY) [9] |

**References**

1. van den Heuvel S, Harlow E. Distinct roles for cyclin-dependent kinases in cell cycle control. Science. 1993;262:2050-4.

2. Rozenblatt-Rosen O, Deo RC, Padi M, Adelmant G, Calderwood MA, Rolland T, et al. Interpreting cancer genomes using systematic host network perturbations by tumour virus proteins. Nature. 2012;487(7408):491-5. doi: 10.1038/nature11288. PubMed PMID: 22810586; PubMed Central PMCID: PMC3408847.

3. Agger K, Cloos PA, Christensen J, Pasini D, Rose S, Rappsilber J, et al. UTX and JMJD3 are histone H3K27 demethylases involved in HOX gene regulation and development. Nature. 2007;449(7163):731-4. Epub 2007/08/24. doi: nature06145 [pii]

10.1038/nature06145. PubMed PMID: 17713478.

4. Sen GL, Webster DE, Barragan DI, Chang HY, Khavari PA. Control of differentiation in a self-renewing mammalian tissue by the histone demethylase JMJD3. Genes Dev. 2008;22(14):1865-70. PubMed PMID: 18628393.

5. Medema RH, Herrera RE, Lam F, Weinberg RA. Growth suppression by p16(ink4) requires functional retinoblastoma protein. Proceedings of the National Academy of Sciences of the United States of America. 1995;92:6289-93.

6. Piboonniyom SO, Timmermann S, Hinds P, Munger K. Aberrations in the MTS1 tumor suppressor locus in oral squamous cell carcinoma lines preferentially affect the INK4A gene and result in increased cdk6 activity. Oral Oncol. 2002;38(2):179-86. PubMed PMID: 11854066.

7. Chen J, Jackson PK, Kirschner MW, Dutta A. Separate domains of p21 involved in the inhibition of Cdk kinase and PCNA. Nature. 1995;374:386-8.

8. Abbas T, Sivaprasad U, Terai K, Amador V, Pagano M, Dutta A. PCNA-dependent regulation of p21 ubiquitylation and degradation via the CRL4Cdt2 ubiquitin ligase complex. Genes Dev. 2008;22(18):2496-506. doi: 10.1101/gad.1676108. PubMed PMID: 18794347; PubMed Central PMCID: PMCPMC2546691.

9. Polyak K, Lee MH, Erdjument-Bromage H, Koff A, Roberts JM, Tempst P, et al. Cloning of p27(Kip1), a cyclin-dependent kinase inhibitor and a potential mediator of extracellular antimitogenic signals. Cell. 1994;78:59-66.
