# Supplementary material for: KDM6A addiction of cervical carcinoma cell lines is triggered by E7 and mediated by p21CIP1 suppression of replication stress
Source: PLoS Pathog. 2017 Oct 2;13(10):e1006661. doi: 10.1371/journal.ppat.1006661 (PMC5638616; doi:10.1371/journal.ppat.1006661)
Supplement: S3 Table — (DOCX) [file ppat.1006661.s003.docx]

**Supplemental Table 3**

| **CDC7 Forward Primer** | 5’ AACTTGCAGGTGTTAAAAAAG 3’ |
| --- | --- |
| **CDC7 Reverse Primer** | 5’ TGAAAGTGCCTTCTCCAAT 3’ |
| **CDT Forward Primer** | 5’ CGGTGGACGAGGTTTCCAG 3’ |
| **CDT Reverse Primer** | 5’ CTGCCGGGGTGGATTTCTT 3’ |
| **DBF4 Forward Primer** | 5’ TGTTTCAGACTAGTGAAGAGAAATC 3’ |
| **DBF4 Reverse Primer** | 5’ AAAAGCCAGTAAATGTAGAAGTTG 3’ |
| **GAPDH Forward Primer** | 5’ GATTTCCACCCATGGCAAATCC 3’ |
| **GAPDH Reverse Primer** | 5’ TGGGATTTCCATTGATGACAAG 3’ |
| **p21^CIP1^ Forward Primer** | 5’ CGATGCCAACCTCCTCAACGA 3’ |
| **p21^CIP1^ Reverse Primer** | 5’ CGCAGACCTCCAGCATCCA 3’ |
| **p27^KIP1^ Forward Primer** | 5’ TGCAACCGACGATTCTTCTACTCAA 3’ |
| **p27^KIP1^ Reverse Primer** | 5’ CAAGCAGTGATGTATCTGATAAACAAGGA 3’ |
